# Community perspectives on epigenetic dementia risk testing: Willingness, implementation preferences, and reasons for not testing in midlife and older adults

**DOI:** 10.1002/alz.71094

**Published:** 2026-02-05

**Authors:** Deirdre M. O'Shea, Devi Dhanekula, Swati Kumar, Lily Wang, Lisa Wiese, Tatjana Rundek, James E. Galvin

**Affiliations:** ^1^ Comprehensive Center for Brain Health Department of Neurology Miller School of Medicine University of Miami Boca Raton Florida USA; ^2^ Miller School of Medicine University of Miami Miami Florida USA; ^3^ Division of Biostatistics Department of Public Health Sciences Miller School of Medicine University of Miami Miami Florida USA; ^4^ Christine E. Lynn College of Nursing Florida Atlantic University Boca Raton Florida USA; ^5^ Department of Neurology and Evelyn F. McKnight Brain Institute Miller School of Medicine University of Miami Miami Florida USA

**Keywords:** Alzheimer's disease, dementia risk, epigenetic testing, health literacy, public attitudes

## Abstract

**INTRODUCTION:**

Epigenetic assays may support non‐invasive dementia risk stratification; community views on willingness and implementation remain under‐characterized.

**METHODS:**

In a survey of 425 adults ≥50 years old, we assessed the willingness for a hypothetical epigenetic test, implementation preferences, reactions to a high‐risk result, behavior‐change intentions, and reasons for not testing using multivariable models.

**RESULTS:**

Overall, 82.1% showed a willingness. Health literacy (odds ratio [OR] = 2.61) and Alzheimer's disease (AD) concern (OR = 2.06) increased that willingness; doctor dependence decreased it (OR = 0.62). The top drivers were perceived to be accuracy and speed. The preferred modality was a combination of biomarker and cognitive over biomarker‐only. Intended changes prioritized alcohol reduction, then diet, exercise, cognitive activity. Risk worry and insurance concerns exceeded stigma; higher literacy related to lower stigma, and epigenetics familiarity and AD worry related to higher insurance concern. The reasons for not testing were data privacy/accuracy concerns, logistics/costs, and needles.

**DISCUSSION:**

Findings support emphasizing test accuracy, turnaround, and governance/legal information when implementing DNAm testing for dementia risk.

## Highlights

- Among older adults, 82% were willing to undergo epigenetic dementia testing.
- Higher health literacy and AD worry increased testing willingness.
- Doctor dependence was linked to a lower willingness to test.
- A combination of biomarker and cognitive testing was the preferred approach.
- Key barriers were data privacy, accuracy doubts, needles, and emotional avoidance.

## 1 INTRODUCTION

Over the past decade, Alzheimer's disease (AD) has emerged as not only a clinical and caregiving challenge but also a major public‐health crisis. Over six million Americans are living with AD and related dementias, with projections nearing 13 million by 2050.^1^ Traditional risk factors such as age, carriage of the apolipoprotein E ε4 allele (*APOE* ε4), and family history, capture only part of an individual's trajectory, prompting researchers to explore epigenetic biomarkers for dementia risk prediction,^2^, ^3^, ^4^ which are considered mitotically heritable yet reversible molecular marks that integrate genetic and environmental influences.^4^, ^5^

The most understood epigenetic mechanism is DNA methylation (DNAm), the covalent addition of a methyl group to cytosine‐phosphate‐guanine (CpG) dinucleotides, which can either repress or facilitate transcription depending on genomic context.^6^ High‐throughput platforms such as array‐based assays and whole‐genome bisulfite sequencing now permit the quantitative interrogation of hundreds of thousands of CpG sites across tissues.^7^ From these data, “epigenetic clocks” (e.g., Horvath, PhenoAge) estimate biological age and have been linked to morbidity, mortality, cognitive decline, and neuroimaging markers of AD pathology.^8^, ^9^ In parallel, targeted methylation panels enriched for immune‐ and inflammation‐related CpGs show promise in predicting conversion from mild cognitive impairment to dementia.^10^, ^11^ Unlike immutable genetic variants, DNA methylation signatures are influenced by modifiable factors (e.g., smoking, diet, stress), which offer dual promise as both risk‐stratification tools and intervention targets.^5^, ^12^ Despite this promise, there is currently no clinically standardized DNAm‐based test for dementia risk, and translation remains in its early stage.^13^

Prior attitude studies focused on genetic testing (e.g., *APOE*) or amyloid/tau blood biomarkers, documenting both enthusiasm for actionable information and concerns about psychological impact, privacy, and insurance discrimination.^14^, ^15^ More recent work examined anticipated reactions to biomarker disclosure among both laypersons and healthcare professionals, highlighting divergent views on utility, interpretability, and ethical implications of early risk information.^16^, ^17^, ^18^ Ethical and policy analyses further underscore the need for robust consent processes and data‐governance frameworks for epigenetic testing.^19^, ^20^, ^21^ However, epigenetics‐specific attitudes remain under‐characterized: Prior surveys largely emphasized genetic or amyloid/tau blood biomarkers rather than DNAm, and few data describe baseline epigenetics knowledge in community samples or how such knowledge relates to testing preferences or intended health behaviors.

To address these gaps, we developed a 20‐min web‐based survey targeting community‐dwelling adults aged 50 years and older. The survey was designed to assess (1) willingness to undergo an epigenetic (DNAm) test for dementia risk; (2) implementation preferences that inform practical workflow design (e.g., perceived accuracy/speed, biospecimen considerations such as blood versus saliva, needle aversion, and data security); (3) anticipated emotional and social consequences, including worry about personal Alzheimer's risk, perceived stigma, insurance concerns, and likelihood of sharing results; and (4) intended behavior change following a hypothetical high‐risk result across diet, physical activity, cognitive engagement, and alcohol use. Since DNAm assays are biospecimen‐based rather than cognitive tests, implementation questions (e.g., specimen type and whether to pair with cognitive screening) are central. Accordingly, our aim was to characterize baseline (unprimed) willingness and implementation preferences in a community sample, situate those preferences within respondents’ epigenetics familiarity, and explore whether familiarity relates to intended health behavior change. We also describe key barriers among those unwilling to test to inform early‐stage education, consent, and workflow planning. This baseline is a prerequisite for designing education/counseling and implementation strategies as the science matures.

## 2 METHODS

### 2.1 Participants

**RESEARCH IN CONTEXT**

1. **Systematic review**: We searched PubMed and Google Scholar (epigenetic testing, DNAm methylation, Alzheimer's disease, public attitudes). Prior work emphasized genetic risk (i.e., *APOE ε4 *) and amyloid/tau biomarker attitudes and disclosure; empirical data on public views of DNAm‐based dementia risk testing, familiarity, and workflow preferences are sparse.
2. **Interpretation**: Among community‐dwelling adults ≥50 years old (*N* = 425), 82.1% were willing to undergo DNAm testing. Higher health literacy and AD worry increased willingness; greater doctor dependence decreased it. Among the willing, perceived accuracy and turnaround time were leading drivers; combined biomarker and cognitive testing was most preferred. After a high‐risk vignette, intended behavior change was strongest for alcohol reduction, then diet, physical activity, and cognitive activities.
3. **Future directions**: Future studies should test education/decision‐aid framing on uptake, refine messaging, and expand to underrepresented groups. Pragmatic studies should assess real‐world uptake, the durability of behavior change, and health impacts and include measures of perceived modifiability/controllability of DNAm risk.

We enrolled community‐dwelling adults aged ≥50 years old without a self‐reported diagnosis of dementia/AD/cognitive impairment. Participants were recruited from (a) the Healthy Brain Initiative (HBI), a longitudinal cohort of racially and ethnically diverse older adults in South Florida^22^; (b) the Rural Vascular Contributions to Cognitive Impairment and Dementia (VCID) study, which focuses on dementia health literacy and vascular risk factors in older adults living in rural and socioeconomically disadvantaged Florida communities surrounding Lake Okeechobee^23^; and (c) the University of Miami Health System “Consent‐to‐Contact” registry. Recruitment aimed for variation in educational attainment and racial/ethnic background to enhance sample diversity. Eligibility criteria were age ≥50, ability to complete an English‐language online survey, and no self‐reported dementia/AD/cognitive impairment. Eligible individuals received an emailed Qualtrics link, provided written online consent under a National Institutes of Health Certificate of Confidentiality, and received a $25 gift card upon completion; the survey required ∼15 to 20 min.

### 2.2 Enrollment flow

Of 1200 invitations, 538 initiated the survey; 503 met eligibility and consented. Of these, 425 provided complete responses to prespecified core items (final analytic sample, *N* = 425). Core items comprised willingness to undergo DNAm testing, demographics, and prespecified psychosocial predictors (e.g., health literacy, AD concern). Non‐core items (e.g., detailed implementation/workflow preferences among the willing, anticipated reactions to a high‐risk result, intended behavior change) were optional and could be skipped. Seventy‐eight participants discontinued the survey before   completing all core items and were therefore excluded from the primary analyses. The University of Miami Institutional Review Board approved all procedures.

### 2.3 Survey development

Survey items were selected a priori informed by the Health Belief Model (HBM), which posits that health‐related behaviors are shaped by perceived susceptibility, perceived barriers, and self‐efficacy^24^ and drew content from the literature on DNAm biomarkers and AD genetic/biomarker attitudes, as well as public attitudes toward genomic testing.^14^, ^15^ A total of 19 items were adapted from Clark et al.’s Alzheimer's biomarker disclosure instrument^16^; wording was harmonized to the epigenetics context and response scales standardized (Table S1 item‐level mapping). For participants indicating willingness to test, we prespecified implementation preferences and practical drivers (e.g., speed, biospecimen/needle aversion, convenience, data security); for those who were unwilling, we prespecified test‐refusal barriers. These lists were derived a priori from previous biomarker‐attitude instruments, common workflow considerations in biomarker implementation, and a targeted review of AD genetic/biomarker‐attitude surveys. By design, these drivers emphasize workflow/practical factors rather than deeper psychosocial motives; a free‐text “other” option captured additional concerns for thematic coding. To estimate baseline (unprimed) community attitudes, we did not provide an educational module before eliciting responses (the potential impact of education is noted in Section 4.7, “Limitations and future directions”). The instrument was pilot‐tested with two cognitively unimpaired HBI participants (≥50 years) for clarity and timing (honorarium $50); pilot data were excluded. The final survey comprised 40 items (two eligibility screeners plus 38 primary) administered in Qualtrics.

### 2.4 Core survey items

#### 2.4.1 Demographics

Age range (50 to 59, 60 to 69, 70 to 79, 80 to 89, 90+), education (less than high school to professional degree), sex (male, female, other), race (select all that apply), Hispanic ethnicity (yes/no), marital status, and family/friend history of cognitive impairment (yes/no/not sure).

#### 2.4.2 Health literacy

Four items were adapted from the Brief Health Literacy Test,^25^ which asked participants to rate the difficulty of understanding written or verbal medical information, confidence in completing medical forms, and need for assistance with medical materials (5‐point scales; reverse‐scored as needed). Self‐rated health and cognition were each measured with a single item assessing perceived overall health and memory/thinking skills (4‐point scale: 1 = poor to 4 = as good as ever). These map to HBM “barriers” (literacy) and “self‐efficacy” (perceived capability to manage health/cognition).^24^

#### 2.4.3 Epigenetics familiarity

Three items: (1) prior awareness of epigenetics (yes/no/don't know), (2) awareness of epigenetic clocks/biological age (yes/no variants), and (3) self‐rated understanding (1 = not well at all to 5 = extremely well). A minimal epigenetics definition was shown within these items to standardize wording without providing an educational primer. “Epigenetics refers to changes in how our genes work that can happen because of our lifestyle, but these changes do not alter the DNA sequence itself.”

#### 2.4.4 Psychosocial beliefs

Three items were included to capture doctor dependence (belief that maintaining health requires physician help), personal agency (responsibility for one's health), and concern about developing AD. Each item was rated on a 5‐point scale (1 = not at all to 5 = a tremendous amount). These map to HBM “self‐efficacy/cues to action” (agency), “barriers” (doctor dependence), and “perceived susceptibility” (AD worry).^24^

#### 2.4.5 Discrimination experiences

Past‐year experiences were captured via a multi‐select item (e.g., gender, race), yielding a count variable (0 to 8). This item indexed potential structural barriers and trust concerns relevant to uptake.

#### 2.4.6 Willingness to take an epigenetic test (primary outcome)

Willingness to undergo a DNAm epigenetic test was assessed with a 4‐point item (1 = definitely not to 4 = definitely yes) and dichotomized for regression analyses (1 = probably/definitely yes vs 0 = definitely/probably not). We deliberately used a four‐category forced‐choice format (definitely/probably yes vs probably/definitely not) to elicit directional inclination in a hypothetical decision context and to reduce midpoint/“no‐opinion” satisficing. Survey‐methods work shows that neutral/no‐opinion options can attract low‐effort respondents and alter marginal distributions without reflecting stable attitudes.^26^, ^27^

#### 2.4.7 Implementation of workflow preferences (optional items)

Among respondents willing to test, we assessed implementation preferences across four options: biomarker only (blood or saliva), cognitive testing only, combined biomarker plus cognitive testing, or neither. Epigenetic risk stratification requires biological material (blood or saliva); thus, this item captured workflow preferences (biological sample alone vs paired with cognitive screening) rather than alternative analytic methods. We included it because blood DNAm profiles and epigenetic aging measures have been linked to AD phenotypes and cognition in prior work, supporting DNAm as a plausible risk stratification tool alongside cognitive screening.^2^, ^3^, ^28^ We also assessed perceived practical facilitators (e.g., accuracy, speed, non‐invasiveness/needle avoidance, convenience, data security). To minimize burden, anxiety/stress and perceived lack of utility were not prespecified; instead, an open “other” option captured additional concerns, which we thematically coded.

#### 2.4.8 Behavior‐change intentions and anticipated reactions

Behavior‐change intentions following a hypothetical high‐risk result were rated (1 = not at all to 5 = extremely) for diet, physical activity, cognitive activities, and alcohol reduction. Anticipated reactions (5‐point scales) included worry about personal AD/dementia risk, concern about insurance impacts, and concern about others’ reactions/stigma. Likelihood of sharing results with a loved one was rated on a 4‐point scale.

#### 2.4.9 Reasons to not test

Participants unwilling to test (defined as “definitely not” or “probably not” respondents) selected barriers (select all that apply), including data‐sharing/privacy concerns, needle aversion, doubt about test accuracy, insurance disclosure, and logistical inconvenience; free‐text (“other”) responses were categorized post‐hoc.

#### 2.4.10 Data handling

All responses were anonymized; payment emails were stored separately and deidentified after processing. Free‐text responses were thematically coded by two raters; discrepancies were resolved by consensus.

### 2.5 Statistical analyses

All analyses were conducted in SPSS version 28. The analytic sample comprised 425 participants who completed all core survey items (willingness to undergo a hypothetical epigenetic test; demographics; psychosocial predictors). Of these, 349 indicated willingness (“probably”/“definitely yes”), and 76 indicated unwillingness (“probably”/“definitely not”). By design, no analytic case had missing core data. Among the willing, optional follow‐up items queried implementation/workflow and modality preferences and anticipated reactions to a high‐risk result; among the unwilling, optional items queried reasons for not testing.

Multi‐item scales were computed as means after reverse‐scoring where required; internal consistency was evaluated with Cronbach's α. Free‐text responses for implementation preferences and reasons for non‐testing were coded thematically by two independent raters, with discrepancies resolved by consensus.

An exploratory factor analysis (EFA) was conducted on 16 core items assessing health literacy, self‐rated health and cognition, epigenetics familiarity, personal agency, doctor dependence, Alzheimer's concern, and discrimination experiences to confirm the underlying construct structure. Principal‐axis factoring with Promax rotation was used. Sampling adequacy was verified by the Kaiser‐Meyer‐Olkin (KMO) measure and Bartlett's test of sphericity; an average KMO of 0.5 to 0.6 is acceptable for samples of 100 to 200.^29^ In our data, KMO = 0.787 and Bartlett's χ^2^(78) = 1,446.39, *p* < 0.001.^30^ Test items included four multi‐item scales including health literacy (four items), self‐rated health and cognition (two items), epigenetics familiarity (three items), and personal agency (two items), as well as three single‐item predictors (doctor dependence, AD concern, discrimination count).

### 2.6 Predictors of test willingness

Hierarchical binary logistic regression models were used to examine predictors of willingness to test. Predictors were entered in five blocks: (1) sociodemographics (age, education, sex, race, Hispanic ethnicity, partnered status), (2) family history of cognitive impairment/dementia, (3) self‐rated health and health literacy, (4) epigenetics familiarity and personal agency, and (5) AD concern, discrimination count, and doctor dependence. All variables were retained. Model fit indices included omnibus χ^2^, −2LL, Cox & Snell and Nagelkerke *R* ^2^, and Hosmer–Lemeshow. Among the 349 willing participants, descriptive statistics summarized implementation preferences (e.g., test accuracy, speed, no needles) and modality preferences (blood/saliva, cognitive tests, both, or neither). Proportions for modality preferences were compared to equal distributions using χ^2^; pairwise tests used z‐tests with Holm correction. A repeated‐measures ANOVA with Greenhouse–Geisser corrections compared behavioral intention (based on if the test indicated high risk for dementia) ratings across four domains: modifying diet (e.g., adopting a Mediterranean diet), increasing physical activity (e.g., walking), engaging in cognitive activities (e.g., puzzles), and reducing alcohol intake, using a 5‐point Likert scale (1 = not at all, 5 = extremely). A second repeated‐measures ANOVA compared three concern types, worry about AD/dementia risk, insurance impacts, and others’ reactions, using a 5‐point Likert scale. Mauchly's test of sphericity was applied, with corrections as needed.

### 2.7 Behavior‐change intentions (within the willing‐to‐test subgroup)

To examine predictors of behavioral/lifestyle changes following a hypothetical post‐test high risk result, we derived a composite behavior‐change intentions score (mean of four 1 to 5 items: diet, physical activity, cognitive activity, alcohol reduction; Cronbach's α = 0.80) and fit a hierarchical multiple linear regression with four blocks entered by theoretical priority, retaining all predictors at each step. Block 1 included sociodemographics: age (50 to 69 [ref], 70 to 79, 80+), sex (male = 1), education (five dummies; < HS [ref]), partnered status (living with a partner = 1), Hispanic ethnicity (yes = 1), race (White = 1), and family history of cognitive impairment (yes = 1). Block 2 (individual‐difference factors): self‐rated health (higher = worse), epigenetics familiarity, personal agency (two‐item scale), doctor dependence, discrimination count, general AD worry, and health literacy. Block 3: intention to disclose results to a loved one. Block 4: concern about others’ reactions (stigma) and concern about insurance impacts. We report *R* ^2^/adjusted *R* ^2^, Δ*R* ^2^ (F‐change), and standardized coefficients (β) for continuous predictors.

### 2.8 Anticipatory reactions following high‐risk test result

We fit parallel hierarchical regressions for four outcomes: (a) post‐result AD/dementia worry (1 to 5), (b) concern about insurance impacts (1 to 5), (c) concern about others’ reactions (stigma; 1 to 5), and (d) intention to disclose results to a loved one (1 to 4). Blocks 1 and 2 matched the behavioral‐intentions model. Block 3 included the behavior‐change intentions composite to test its incremental association with psychosocial reactions. Reporting conventions matched those above.

For the 76 unwilling to test participants, descriptive statistics summarized barriers to testing (e.g., data‐sharing concerns, needle aversion), with frequencies reported for each reason.

### 2.9 Sensitivity analyses examined missing responses to optional items

We examined missing responses to optional items among the willing to test group (*n* = 349). Bivariate associations with missingness used cross‐tabulations, Welch's *t*‐tests, and logistic regression. Significant bivariate predictors were entered into a multivariable logistic model (outcome: missing vs non‐missing).

## 3 RESULTS

The sample was diverse in age, education, and race/ethnicity, with 77.4% aged 50 to 69 years, 36.7% holding bachelor's degrees, 23.8% identifying as Hispanic, 23.4% identifying as Black, and 5.9% as Other (Table 1). Over half of participants (59.5%) reported a family history of cognitive impairment/dementia. Overall, 349/425 (82.1%) respondents indicated they would undergo an epigenetic (DNAm) test for dementia risk (“definitely yes” or “probably yes”). The four‐level distribution of willingness to undergo testing was as follows: definitely yes = 43.3% (184/425), probably yes = 38.8% (165/425), probably not = 12.2% (52/425), and definitely not = 5.6% (24/425).

**TABLE 1:** Participant characteristics (*N* = 425).

Characteristic	*n* (%) or M (SD)	Range
Age, years		
50 to 69	329 (77.4)	
70 to 79	72 (16.9)	
80+	23 (5.4)	
Education		
High school or less	40 (9.4)	
Some college/associate's	81 (19.1)	
Bachelor's	156 (36.7)	
Master's	107 (25.2)	
Doctorate/professional	40 (9.4)	
Sex		
Male	242 (56.9)	
Female	180 (42.4)	
Other	3 (0.7)	
Race		
White	301 (70.8)	
Black	99 (23.3)	
Other	25 (5.9)	
Hispanic ethnicity		
Non‐Hispanic	324 (76.2)	
Hispanic	101 (23.8)	
Marital status		
Single	37 (8.7)	
Married	274 (64.5)	
Living with partner	19 (4.5)	
Divorced/separated	39 (9.2)	
Widowed	30 (7.1)	
Never married	1 (0.2)	
Multiple/no response	25 (5.9)	
Family history of cognitive impairment		
Yes	253 (59.5)	
No	141 (33.2)	
Not sure	31 (7.3)	
Psychosocial scales		
Self‐rated health/cognition	3.01 (0.60)	1.00 to 4.00
Epigenetics familiarity	2.18 (0.86)	1.00 to 4.50
Personal agency (two items)	3.79 (0.81)	1.50 to 5.00
Doctor dependence	2.80 (1.12)	1.00 to 5.00
Health literacy	3.66 (1.00)	1.25 to 5.00
Discrimination count	1.37 (1.53)	0.00 to 8.00
Alzheimer's concern	2.98 (1.11)	1.00 to 5.00
Willingness to take epigenetic test	349 (82.1)	

### 3.1 Awareness of epigenetics

Recognition of epigenetics terms was mixed. For “heard of epigenetics,” 50.0% (212/425) reported yes and 44.2% (188/425) no; 5.6% (24/425) selected “don't know.” For “heard of epigenetic clocks/biological age,” 37.2% (158/425) had heard of both, 13.4% (57/425) clocks only, 12.7% (54/425) biological age only, and 36.7% (156/425) said no. Self‐rated understanding was 29.6% not well at all, 26.6% slightly, 28.7% moderately, 10.4% very, and 4.7% extremely.

### 3.2 Exploratory factor analyses

Results from the EFA on 16 core items confirmed four multi‐item scales: health literacy (α = 0.83), self‐rated health and cognition (α = 0.73), epigenetics familiarity (α = 0.51), and personal agency (α = 0.64) and three single‐item predictors (doctor dependence, Alzheimer's concern, discrimination count). Sampling adequacy was acceptable (KMO = 0.787), and Bartlett's test indicated sufficient correlations among items for factor analysis (χ^2^[78] = 1,446.39, *p* < 0.001). These results supported the suitability of the data structure for factor analysis, although the relatively low alpha for epigenetics familiarity (α = 0.51) suggested limited internal consistency.

### 3.3 Predictors of willingness

Statistically significant predictors from hierarchical binary logistic regression are summarized in Table 2, with coefficients for all variables provided in Supplementary Table 2S. The final model demonstrated acceptable fit (−2LL = 300.77, Cox & Snell *R* ^2^ = 0.18, Nagelkerke *R* ^2^ = 0.30, Hosmer–Lemeshow χ^2^[8] = 3.633, *p* = 0.889). Higher health literacy (odds ratio [OR] = 2.61, 95% CI [1.73, 3.95], *p* < 0.001) and greater concern about developing AD (OR = 2.06, 95% CI [1.48, 2.85], *p* < 0.001) increased the odds of willingness, whereas stronger belief in doctor dependence decreased the odds (OR = 0.615, 95% CI [0.45, 0.84], *p* = 0.002). Demographics, family history, self‐rated health, epigenetics familiarity, personal agency, and discrimination were not significant predictors.

**TABLE 2:** Final logistic regression predicting willingness to take an epigenetic test.

Predictor	B	SE	Wald	OR	95% CI	*p*
Health literacy	0.960	0.211	20.74	2.61	[1.73, 3.95]	< 0.001
Alzheimer's concern	0.722	0.167	18.77	2.06	[1.48, 2.85]	< 0.001
Doctor dependence	−0.486	0.156	9.73	0.62	[0.45, 0.84]	0.002

*Note*: –2 log likelihood = 300.77; Cox & Snell R^2^ = 0.18; Nagelkerke R^2^ = 0.30. Only statistically significant predictors are shown here. All models adjusted for demographics (age, education, sex, race, Hispanic ethnicity, and marital status) as well as other psychosocial variables; none of these were significant. Full coefficients, including non‐significant predictors, are available in Supplementary Table 1S.

### 3.4 Implementation preferences/drivers

Among the 349 participants willing to undergo testing, 292 provided complete responses on implementation preferences (Table 3). The most endorsed reasons were belief in test accuracy (64.4%) and speed compared to cognitive/memory testing (56.5%). Fewer participants prioritized non‐invasiveness (17.5% saliva only, 11.6% blood only, 17.5% no needles). Free‐text responses (3.1%) included early detection, altruism, and last‐resort options. In terms of modality/workflow preferences, 49.0% endorsed both cognitive testing and biomarker assays, 31.5% selected biomarker only, 18.5% selected cognitive tests only, and 1.0% selected neither. The distribution of preferred implementation modality differed from equal proportions, χ^2^(3) = 144.1, *p* < 0.001. Pairwise tests showed combined > biomarker‐only (*z* = 3.33, *p* = 0.0009), combined > cognitive‐only (*z* = 6.34, *p* < 1×10^−9^), combined > neither (*z* = 11.59, *p* < 1×10^−30^); biomarker‐only > cognitive‐only (*z* = 3.15, *p* = 0.0016) and > neither (*z* = 9.14, *p* < 1×10^−19^); cognitive‐only > neither (z = 6.75, p≈1×10^−11^). All pairwise results remain significant after Holm correction.

**TABLE 3:** Implementation preferences for epigenetic testing among those participants willing to test (*n* = 292 provided responses).

Reason	*n*	Percentage of willing respondents
Believe it would be accurate	188	64.4
Test would be quick versus cognitive/memory testing	165	56.5
Only if it did not involve needles (saliva only)	51	17.5
Only if it used saliva (not blood)	34	11.6
Only if it used blood (not saliva)	32	11
Other (please specify)	9	3.1

*Note*: Only those indicating willingness (*n* = 349) were asked about implementation preferences; 292 provided responses.

### 3.5 Behavior‐change intentions

Willing‐to‐test participants reported moderate to high intentions to change behaviors if faced with a high‐risk test result. A repeated‐measures ANOVA revealed a significant effect of behavioral domain, F(2.71, 784.67) = 4.12, *p* = 0.009 with intentions to reduce alcohol intake (M = 4.05, SD = 1.04, 70.2% rated 4 or 5) significantly stronger than intentions to increase exercise (M = 3.88, SD = 0.95, 65.7%; *p* = 0.046) or engage in cognitive activities (M = 3.87, SD = 0.95, 66.3%; *p* = 0.050). Intentions to modify diet were also high (M = 3.88, SD = 1.01, 67%). A second ANOVA compared concerns, revealing a significant effect of concern type, F(1.95, 566.53) = 15.91, *p* < 0.001.

### 3.6 Predictors of behavior‐change intentions

The final model was significant: *R* ^2^ = 0.527, adj. *R* ^2^ = 0.490, F(21, 267) = 14.16, *p* < 0.001. Higher personal agency (β = 0.405, *p* < 0.001), higher health literacy (β = 0.242, *p* < 0.001), stronger intention to disclose to a loved one (β = 0.184, *p* < 0.001), family history (β = 0.164, *p* < 0.001), worse self‐rated health (β = 0.155, *p* = 0.006), and greater general AD worry (β = 0.149, *p* = 0.004) were associated with higher behavior‐change intentions. Stigma concern was also positively associated (β = 0.134, *p* = 0.010). Insurance concern trended positive (β = 0.096, *p* = 0.060). Age, sex, education, partnered status, Hispanic ethnicity, race (White), epigenetics familiarity, doctor dependence, and discrimination were not significant. Results are shown in Table 4.

**TABLE 4:** Predictors of behavioral change following a high‐risk test result.

Predictor	B	SE	β	*t*	*P*
Intercept	0.025	0.366	—	0.069	0.945
Age 70 to 79 (ref 50‐69)	0.135	0.103	0.059	1.309	0.192
Age 80+ (ref 50‐69)	0.161	0.162	0.044	0.998	0.319
Male (vs female)	0.088	0.075	0.055	1.175	0.241
High school graduate (ref < HS)	−0.112	0.142	−0.055	−0.786	0.432
Some college	−0.039	0.135	−0.025	−0.292	0.77
Bachelor's degree	0.094	0.142	0.053	0.663	0.508
Graduate degree	−0.061	0.175	−0.022	−0.349	0.727
Partnered (married/living with partner = 1)	0.104	0.121	0.04	0.859	0.391
Hispanic (yes = 1)	−0.087	0.088	−0.048	−0.996	0.32
White (vs all others)	−0.007	0.078	−0.004	−0.083	0.934
Family history (yes = 1)	0.262	0.07	0.164	3.725	<0.001
Self‐rated health (higher = worse)	0.155	0.056	0.133	2.779	0.006
Epigenetics familiarity	−0.064	0.046	−0.072	−1.403	0.162
Personal agency (two‐item)	0.4	0.048	0.405	8.303	<0.001
Doctor dependence	−0.043	0.036	−0.058	−1.195	0.233
Discrimination count	−0.024	0.023	−0.049	−1.034	0.302
General AD worry	0.105	0.036	0.149	2.943	0.004
Health literacy	0.196	0.044	0.242	4.462	<0.001
Intend to disclose to loved one	0.179	0.046	0.184	3.904	<0.001
Concern – others’ reactions (stigma)	0.085	0.033	0.134	2.593	0.01
Concern – insurance impacts	0.065	0.034	0.096	1.893	0.06

*Note*: Outcome = mean intention (1 to 5; higher = stronger intention). “Education levels” are dummy indicators, with the lowest education category as reference. Partnered indicates married or living with a partner. Family history coded 1 = yes, 0 = no or not sure. Self‐rated health coded higher = worse. Race coded White versus all others (1/0). Coefficients are unstandardized B with standard errors; β are standardized.

### 3.7 Anticipated reactions following a high‐risk test result

Worry about AD/dementia risk was moderate to high (M = 3.55, SD = 0.99), with 55.1% of participants reporting high worry (ratings of 4 or 5). Concern about insurance impacts was similar (M = 3.48, SD = 1.14; 51.4% high concern), and both were significantly greater than concern about others’ reactions (M = 3.16, SD = 1.23; 38.0% high concern; both *p*s < 0.001), with no statistically significant difference between risk worry and insurance concern.

### 3.8 Predictors of anticipated reactions (fully adjusted models)

#### 3.8.1 Stigma

Higher general AD concern (β = 0.233, *p* < 0.001) and living with a partner/married (β = 0.115, *p* = 0.045) were associated with greater stigma concern; compared with < HS, some college/AA (β = 0.250, *p* = 0.017) and graduate degree (β = 0.208, *p* = 0.008) were also significant. Higher health literacy was associated with lower stigma concern (β = −0.139, *p* = 0.039). All other covariates were not significant.

#### 3.8.2 Insurance concern

Greater familiarity with epigenetic biomarkers (β = 0.167, *p* = 0.011) and greater general AD concern (β = 0.163, *p* = 0.010) were associated with higher insurance concern. Being age 80+ (vs 50 to 69; β = −0.142, *p* = 0.013) and White race (vs non‐White; β = −0.147, *p* = 0.018) were associated with lower concern. Other predictors were not significant.

#### 3.8.3 Post‐test AD worry

Greater agency (β = 0.244, *p* < 0.001) and higher general AD concern (β = 0.250, *p* < 0.001) predicted more post‐test worry. All other covariates were not significant.

#### 3.8.4 Disclosure to a loved one

Greater agency (β = 0.259, *p* < 0.001), age 70 to 79 (vs 50 to 69; β = 0.123, *p* = 0.036), and living with a partner (β = 0.169, *p* = 0.005) predicted higher disclosure likelihood. All other covariates were not significant.

#### 3.8.5 Reasons for not testing

Among the 76 unwilling participants, reasons for unwillingness to test were concern about data sharing (41.1%), needle aversion (31.5%), and doubt about test accuracy (27.4%). Concerns about insurance disclosure (26.0%) and inconvenience of doctor's visits (23.3%) were also notable. Free‐text “other” responses (*n* = 5 participants; 11 coded comments) revealed additional themes: cost concerns (3/11; 27.3%), emotional avoidance/anxiety (e.g., “don't want to know”) (2/11; 18.2%), and perceived lack of need/utility (e.g., “I don't see the need to know”) (2/11; 18.2%). Single comments reflected uncertainty about data completeness/representativeness (“do people that look like me participate?”).

#### 3.8.6 Sensitivity analyses of missingness

Among those willing to test, *n* = 349, responses to behavioral intent and anticipatory reactions items was 83.7% (292/349). Non‐completion was non‐random: it was higher in ages 70 to 79 (38.5%) and 80+ (21.1%) versus 50 to 69 (10.6%; χ^2^[2] = 30.05, *p* < 0.001), higher in females (26.3%) versus males (8.6%; χ^2^[1] = 19.64, *p* < 0.001), and differed by race (White 19.4%, Black 3.9%, Other 25.0%; χ^2^[2] = 11.34, *p* = 0.003; small “Other” cell). Although optional‐item non‐response was systematically related to health literacy, sex, and race, these covariates are included in all multivariable outcome models, mitigating risk of bias. Missingness did not differ by Hispanic ethnicity, education, marital status, or family history. On continuous measures, skippers reported lower AD concern, lower epigenetics familiarity, better self‐rated health, substantially higher health literacy, fewer discrimination events, and lower doctor dependence (all *p* ≤ 0.023), with no difference in agency. We also examined a multivariate model of significant bivariate predictors of missingness using logistic regression and missing and non‐missing as the outcome. Higher health literacy was the dominant predictor of non‐completion (OR = 12.06, 95% CI: 4.63 to 31.45, *p* < 0.001), and female sex was also associated with higher odds (OR = 2.79, 95% CI: 1.22 to 6.41, p = 0.016), and Black (vs White) race with lower odds (OR = 0.17, 95% CI: 0.04 to 0.67, *p* = 0.012). Age showed a borderline effect (70 to 79 vs 50 to 69: OR = 2.18, *p* = 0.071), while familiarity with epigenetics, self‐rated health, discrimination count, AD concern, and doctor dependence were not independently associated after adjustment. These findings corroborate bivariate patterns and indicate that optional‐item non‐response is systematically related to health literacy, sex, and race

## 4 DISCUSSION

Most adults aged 50+ (82.1%) expressed a willingness to undergo a hypothetical DNAm‐based blood or saliva test. Willingness was higher with greater health literacy (2.6×) and AD concern (2.1×) and lower with stronger doctor dependence (−38%). Implementation preferences emphasized accuracy and speed. Among willing respondents, combined biomarker and cognitive testing was most common (49.0%), followed by biomarker‐only (31.5%); cognitive‐only (18.5%) and neither (1.0%) were least common. After a high‐risk result, intended behavior change was strongest for alcohol reduction, then diet, exercise, and cognitive activities. In fully adjusted models, personal agency, health literacy, disclosure intention, family history, worse self‐rated health, and general AD worry predicted stronger behavior‐change intentions; demographic factors did not remain independent predictors. Concerns about insurance discrimination were more common than stigma concerns. Overall, the study provides epigenetics‐specific estimates, familiarity distributions, and workflow preferences linked to behavior change.

### 4.1 Contextualizing willingness within broader biomarker attitude research

The high willingness rate aligns with population polling that reports strong openness to simple blood‐based AD tests (e.g., 91% to 95% in a recent US report) and substantial interest in learning dementia risk in United Kingdom (UK) samples.^31^, ^32^ Our results suggest that enthusiasm extends to blood‐ or saliva‐based epigenetic testing, a modality framed here as risk stratification rather than pathology detection. Health literacy emerged as the strongest independent predictor of willingness, consistent with evidence that better literacy supports engagement with preventive screening and novel tools.^33^, ^34^ AD concern was also a significant predictor of willingness, consistent with prior work showing that perceived susceptibility motivates test interest.^35^, ^36^ Lower willingness among participants with higher doctor dependence may reflect reduced health autonomy, a pattern previously noted in genetic testing contexts.^37^ Together, these findings suggest that education and decision aids that enhance comprehension and autonomy may support equitable uptake.

National polling of US adults aged 65 to 80 found low familiarity with blood‐based biomarkers (BBMs) for AD (≈19% at least somewhat familiar) and limited immediate interest in testing (≈9% would test now), alongside concerns about reliability, privacy, and potential distress/stigma.^37^ UK Dementia Attitudes Monitor data similarly show strong receptivity to non‐invasive blood tests and reluctance toward lumbar puncture, mirroring our respondents’ preference for low‐burden modalities.^32^ The 2025 Alzheimer's Disease Facts & Figures report documents high belief in early detection and interest in simple diagnostic tests, consistent with our high in‐principle willingness rates.^31^ In our sample, epigenetics awareness (term recognition after a brief definition) was higher than BBM familiarity in national polls, but constructs differ (clinical‐test familiarity vs term recognition), so comparisons should be cautious. Our higher hypothetical willingness likely reflects differences in framing, immediacy, and the brief definition provided at item onset.^26^

Findings from trial and registry studies also suggest that, despite high stated interest, some participants decline learning personal biomarker results due to anxiety, uncertain utility, or downstream implications, and real‐world uptake can lag stated interest (intent–behavior gap).^38^, ^39^ The REVEAL trials (*APOE* ε4 risk disclosure) generally found no major short‐term psychological harms, but that educational framing can temper test interest,^14^, ^15^ a point we incorporate explicitly as a limitation below. Thus, our results complement pathology‐oriented surveys by providing epigenetics‐specific estimates and implementation preferences.

### 4.2 Implementation/workflow preferences

Among willing respondents, the most frequently endorsed implementation drivers were belief in test accuracy and speed relative to cognitive/memory testing, whereas fewer participants emphasized non‐invasiveness. Population polls showing that concerns about reliability and a desire for simple, low‐burden tests shape attitudes toward blood‐based dementia testing.^40^, ^41^ Accordingly, early implementation should emphasize clear communication about analytic validity and expected turnaround times while offering saliva options for needle‐averse subgroups noted in prior surveys.^42^ Regarding modality/workflow preferences, nearly half selected a combined approach (biomarker and cognitive testing), followed by biomarker‐only, with cognitive‐only and neither being least common. Although exploratory, these data highlight that most respondents preferred an integrated approach combining biological and cognitive information, consistent with current research frameworks recommending multimodal risk evaluation,^43^ and are practically useful for planning early implementation (e.g., whether to co‐schedule cognitive screening with phlebotomy).

### 4.3 Behavioral change intentions following a high‐risk test result

Following a high‐risk epigenetic test result, most participants intended to adopt at least one healthy behavior, consistent with prior research.^16^, ^44^ In our sample, intentions were strongest for alcohol reduction, followed by dietary modification, physical activity, and cognitive activities. This ordering contrasts somewhat with the ranking reported in prior work, where dietary and cognitive changes were prioritized over alcohol reduction. While alcohol use contributes modestly to dementia risk compared to inactivity,^45^ it may be perceived as easier to modify, in contrast to the sustained effort required for exercise or cognitive training, as emphasized in multidomain trials (e.g., FINGER).^46^ Unlike static genetic markers, DNAm biomarkers can reflect lifestyle impacts over time,^47^, ^48^ making them especially attractive for ongoing risk monitoring and prevention.

### 4.4 Predictors of behavioral intentions following a high‐risk test result

Consistent with the HBM and Social Cognitive Theory, post‐result behavioral intentions were most strongly related to personal agency and health literacy, with additional contributions from general AD worry, stigma, family history, and worse self‐rated health. These correlates map onto capability/motivation constructs documented in dementia prevention and risk‐disclosure studies.^24^, ^33^, ^35^, ^36^ Education and partnering advantages attenuated after adjustment, consistent with effects operating through literacy, agency, and disclosure intent.^33^, ^34^, ^49^ Group differences by ethnicity were not independent predictors in the final model, implying that modifiable factors (literacy, agency, trust) may account for disparities often attributed to demographic categories.^50^, ^51^

### 4.5 Anticipated reactions following a high‐risk test result

Anticipated reactions following a high‐risk result. Risk worry and insurance concern were both more frequently endorsed than concern about others’ reactions, with no meaningful difference between risk worry and insurance concern. In fully adjusted models, general AD worry was a common correlate across outcomes; stigma concern was higher with greater AD worry, partnered status, and some college/graduate education and lower with higher health literacy, consistent with prior studies.^37^, ^52^ Insurance concern was higher with greater epigenetics familiarity and AD worry and lower among adults 80 and older and White participants, plausibly reflecting differential exposure to/understanding of coverage protections (e.g., Medicare at older ages) and persistent anxieties about non‐health lines (life/disability) despite the Genetic Information Nondiscrimination Act (GINA).^53^, ^54^, ^55^ Post‐test worry was higher with agency and AD worry, and disclosure intent was higher with agency, age 70 to 79, and partnered status, indicating that engaged, planful respondents may also anticipate higher emotional load, an effect seen in disclosure trials.^14^, ^15^ Implementation should emphasize clear communication on analytic validity, data governance, and the scope/limits of legal protections (e.g., GINA for health insurance vs life/disability), with optional supports for shared disclosure.

### 4.6 Reasons for being unwilling to test

Among unwilling participants, leading concerns were data privacy and accuracy; logistical burden and costs were also cited. Needle aversion underscores the value of saliva options. Despite GINA protections for health insurance/employment, anxieties about non‐health lines (life/disability) and data use persist.^55^, ^56^, ^57^, ^58^ Emotional avoidance and perceived lack of need/utility, well described in dementia contexts, also emerged.^37^, ^40^, ^58^, ^59^ Together, these results highlight that hypothetical interest in epigenetic testing is shaped not only by beliefs about accuracy or invasiveness but also by broader concerns about autonomy, access, and trust. Addressing these concerns is essential for equitable implementation.

### 4.7 Limitations and future directions

This study has important limitations. First, the hypothetical survey design may overestimate real‐world uptake, and we did not observe actual testing behavior. Second, the survey was intentionally unprimed (no education/decision‐aid module) to approximate early implementation, but education can temper willingness to test^58^; future trials should randomize educational/decision‐aid exposure. Third, our four‐category willingness item lacked a neutral option, which may shift some fence‐sitters into adjacent categories^26^, ^27^; future versions should include a midpoint or staged vignettes. Fourth, item design and measurement choices constrain interpretation: select outcomes (e.g., general AD worry vs post‐result worry) were asked of different denominators; several motivators/barriers were captured via free‐text rather than as fixed response options; and perceived modifiability/controllability of DNAm risk was not measured, limiting mechanistic inference. Fifth, the sample skewed toward more educated, non‐Hispanic White respondents and required internet access, limiting generalizability; all measures were self‐reported and thus vulnerable to non‐response and social‐desirability biases. Future work should evaluate actual uptake in more diverse populations, include baseline behavioral data, prespecify psychosocial barriers alongside workflow drivers, measure perceived modifiability/controllability, and test supportive interventions that target literacy, agency, and data‐governance understanding.

## 5 CONCLUSIONS

This study provides one of the first empirical assessments of public attitudes toward epigenetic testing for dementia risk. Distinct from pathology‐oriented BBM surveys, it reports epigenetics‐specific familiarity, workflow preferences (blood vs saliva; with/without cognitive screening), and links to intended behavior change. Adults aged 50+ expressed a high willingness to test, particularly those with greater health literacy and concern about AD. Implementation drivers centered on perceived accuracy and speed, and behavior‐change intentions were strong, especially for alcohol reduction. Equitable deployment will require clear, plain‐language communication, low‐burden procedures (including saliva options), and culturally responsive engagement that addresses privacy, cost, and perceived utility.

## CONFLICT OF INTEREST STATEMENT

The authors declare that they have no conflicts of interest related to the content of this manuscript. Author disclosures are available in the Supporting Information.

## CONSENT STATEMENT

All participants provided written informed consent prior to participation in the study under a Certificate of Confidentiality from the NIH.

## Supporting information



Supporting information

Supporting information

Supporting information
