# Supplementary material for: Community perspectives on epigenetic dementia risk testing: Willingness, implementation preferences, and reasons for not testing in midlife and older adults
Source: Alzheimers Dement. 2026 Feb 5;22(2):e71094. doi: 10.1002/alz.71094 (PMC12875745; doi:10.1002/alz.71094)
Supplement: Supplementary file 1 — Supporting information [file ALZ-22-e71094-s001.docx]

Table S1. Item mapping between the Epigenetics-Based Survey (EBS) and the Clark instrument

| **A. Direct or modified/collapsed adaptations (n = 19)** | | |
| --- | --- | --- |
| **Clark item (ID & stem)** | **EBS survey item (Q# & stem)** | **Adaptation note** |
| q3 --- Willing to enroll in "brain marker" study (learn results) | Q17 --- "Would you take an epigenetic (DNAm) blood/saliva test for AD/dementia risk?" | Direct construct; context shifted from study-enrollment to DNAm test uptake; 5-pt→4-pt willingness. |
| q6--q8 --- Willingness by method: PET, LP/CSF, blood | Q18 --- Preferred workflow: biomarker only vs cognitive only vs both vs neither | Collapsed across methods into workflow preference (biological sample ± cognitive screening). |
| q14f --- "How worried would you be about your risk?" | Q20 --- "How worried would you be about risk if high-risk DNAm result?" | Direct; same 5-pt worry. |
| q14k --- Concern about insurance impacts | Q21 --- "How concerned about insurance impacts if high-risk result?" | Direct. |
| q14m --- Concern others would act differently (stigma) | Q22 --- "Concern about others' reactions if high-risk result?" | Direct. |
| q15 --- Likelihood of telling a loved one | Q23 --- "How likely to tell a loved one?" | Direct; same 4–5 pt likelihood. |
| q17a --- Behavior change: diet | Q24 --- "Likelihood to change diet" | Direct. |
| q17b --- Behavior change: physical activity | Q25 --- "Likelihood to increase physical activity" | Direct. |
| q17f --- Behavior change: cognitive activities | Q26 --- "Likelihood to engage in cognitive activities" | Direct. |
| q17g --- Behavior change: reduce alcohol | Q27 --- "Likelihood to reduce/eliminate alcohol" | Direct. |
| q21 --- General concern about getting AD | Q31 --- "How concerned are you that you will develop AD/dementia?" | Direct. |
| q25d --- Belief you can lower AD risk | Q32 --- "How much can you do to lower your risk?" | Direct. |
| q25a --- "Can only stay healthy with doctors" (doctor dependence) | Q28 --- "You can only stay healthy with help from doctors" | Direct. |
| q25b --- "You are directly responsible for your health" | Q29 --- "You are directly responsible for your health" | Direct. |
| q28 --- Self-rated overall health | Q9 --- "How would you rate your overall health?" | Direct (scale phrasing aligned). |
| q29 --- Self-rated memory | Q10 --- "How would you rate your memory/thinking?" | Direct (domain broadened to memory/thinking). |
| q12 --- Ever had genetic (APOE) test | Q11 --- "Ever had a genetic test for AD risk (APOE)?" | Direct. |
| q12a--q12b --- Learned APOE result; higher risk | Q13 --- "Were you told you had APOE-ε4 / higher risk?" | Direct (combined). |
| q27 --- Reasons for discrimination (check all) | Q33 --- Discrimination reasons (check all) | Direct (frequency battery omitted; reasons retained). |
| **B. Conceptually informed (not counted in the 19)** | | |
| **Clark reference (construct)** | **Your survey item (Q# & stem)** | **Note on relationship** |
| q4a (open) "reasons for willingness"; q9a--h (importance of reasons to learn results) | Q19 --- "Reasons I would take the DNAm test" (accuracy, speed, needles, other; check-all) | You converted open/importance content into a pragmatic checklist tailored to DNAm workflows. |
| q4b (open) "concerns about enrolling" | Q30 --- "Reasons I would not take the test" (accuracy doubt, doctor visit burden, needles, data sharing, insurance, other) | Structured checklist derived from Clark's open-ended concerns. |
| q19--q20 (exposure/caregiving) | Q3 --- "Family/friend ever diagnosed with cognitive impairment/Alzheimer's?" | Related exposure construct; different operationalization. |
| --- | Q12 --- Sample type provided for prior genetic test (saliva/finger-stick) | New DNAm-relevant operational detail (not in Clark). |
| --- | Q14--Q16 --- Epigenetics familiarity & understanding (heard of epigenetics/clocks; self-rated understanding) | New constructs specific to DNAm. |
| --- | Q8 --- Perceived representation of one's ethnic community in brain health research | New item on representation/trust climate; no Clark analogue. |
| --- | Q4--Q7 --- Health literacy (4 items) | New scale; not present in Clark instrument. |

Note. This table shows how items in the newly developed Epigenetics-Based Survey (EBS) map to items from the Clark et al. instrument. The mapping is divided into two sections: (A) items that were directly adapted or modified/collapsed from Clark items (n=19), and (B) items that were conceptually informed by Clark constructs but are not 1:1 mappings. For Section A, the table provides the original Clark item ID and abbreviated stem, the corresponding EBS item number and abbreviated stem, and notes on how the item was adapted. Section B provides conceptual mappings where EBS items were informed by but differ substantially from Clark items, or represent entirely new constructs specific to DNA methylation (DNAm) testing. Wording is abbreviated for space constraints.
