# Supplementary material for: Community perspectives on epigenetic dementia risk testing: Willingness, implementation preferences, and reasons for not testing in midlife and older adults
Source: Alzheimers Dement. 2026 Feb 5;22(2):e71094. doi: 10.1002/alz.71094 (PMC12875745; doi:10.1002/alz.71094)
Supplement: Supplementary file 2 — Supporting information [file ALZ-22-e71094-s003.docx]

Table S2. Full binary logistic regression model predicting willingness to take DNA methylation test for Alzheimer's disease risk

| **Predictor** | **B** | **S.E.** | **Wald** | **df** | **Sig.** | **Exp(B)** | **95% C.I. for EXP(B)** | |
| --- | --- | --- | --- | --- | --- | --- | --- | --- |
|  |  |  |  |  |  |  | **Lower** | **Upper** |
| **Age group (overall)** |  |  | 3.925 | 2 | 0.14 |  |  |  |
| 70–79 vs 50–69 | 0.912 | 0.524 | 3.032 | 1 | 0.082 | 2.488 | 0.892 | 6.943 |
| 80+ vs 50–69 | -0.557 | 0.723 | 0.593 | 1 | 0.441 | 0.573 | 0.139 | 2.364 |
| **Sex (overall)** |  |  | 0.002 | 2 | 0.999 |  |  |  |
| Female vs Male | -0.016 | 0.333 | 0.002 | 1 | 0.962 | 0.984 | 0.512 | 1.89 |
| Other vs Male | -23.418 | 24164.32 | 0 | 1 | 0.999 | 0 | 0 | . |
| **Race (overall)** |  |  | 1.819 | 2 | 0.403 |  |  |  |
| Black vs White | -0.471 | 0.352 | 1.792 | 1 | 0.181 | 0.624 | 0.313 | 1.244 |
| Other race vs White | -0.012 | 0.789 | 0 | 1 | 0.988 | 0.989 | 0.21 | 4.643 |
| **Education (overall)** |  |  | 6.675 | 4 | 0.154 |  |  |  |
| Some college/Associate's vs High school or less | 0.218 | 0.627 | 0.12 | 1 | 0.729 | 1.243 | 0.364 | 4.248 |
| Bachelor's vs High school or less | -0.714 | 0.595 | 1.441 | 1 | 0.23 | 0.49 | 0.153 | 1.571 |
| Master's vs High school or less | -0.109 | 0.654 | 0.028 | 1 | 0.868 | 0.897 | 0.249 | 3.231 |
| Doctorate/Professional vs High school or less | -0.896 | 0.7 | 1.635 | 1 | 0.201 | 0.408 | 0.103 | 1.612 |
| Hispanic vs Non-Hispanic | 0.485 | 0.389 | 1.551 | 1 | 0.213 | 1.624 | 0.757 | 3.481 |
| Married/living with partner vs Not married | -0.327 | 0.374 | 0.764 | 1 | 0.382 | 0.721 | 0.347 | 1.501 |
| Family history of dementia (Yes vs No) | 0.351 | 0.306 | 1.321 | 1 | 0.25 | 1.421 | 0.781 | 2.587 |
| Self-rated health | 0.039 | 0.23 | 0.029 | 1 | 0.865 | 1.04 | 0.663 | 1.632 |
| **Health literacy** | **0.96** | 0.211 | 20.739 | 1 | **<.001** | **2.612** | 1.728 | 3.949 |
| Familiarity with epigenetics | -0.215 | 0.203 | 1.119 | 1 | 0.29 | 0.807 | 0.542 | 1.201 |
| Agency (2-item scale) | 0.18 | 0.197 | 0.84 | 1 | 0.359 | 1.198 | 0.814 | 1.761 |
| **Concern about developing Alzheimer's disease** | **0.722** | 0.167 | 18.776 | 1 | **<.001** | **2.058** | 1.485 | 2.853 |
| Perceived discrimination (count) | 0.051 | 0.11 | 0.213 | 1 | 0.645 | 1.052 | 0.848 | 1.306 |
| **Doctor dependence** | **-0.486** | 0.156 | 9.725 | 1 | **0.002** | **0.615** | 0.453 | 0.835 |
| Constant | -2.324 | 1.404 | 2.74 | 1 | 0.098 | 0.098 |  |  |

Note. This table presents the complete results of a binary logistic regression model examining predictors of willingness to take a DNA methylation (DNAm) test for Alzheimer's disease/dementia risk assessment. The outcome variable is a binary indicator of willingness (willing vs. not willing). Predictor variables include demographic characteristics (age group, sex, race, education, Hispanic ethnicity, marital status), family history of dementia, self-rated health, health literacy, familiarity with epigenetics, agency beliefs (2-item scale), concern about developing Alzheimer's disease, perceived discrimination experiences (count), and doctor dependence beliefs. For categorical predictors, the reference category is indicated (e.g., "Female vs Male" indicates Male is the reference). The table reports unstandardized regression coefficients (B), standard errors (S.E.), Wald chi-square statistics, degrees of freedom (df), statistical significance (Sig.), odds ratios (Exp(B)), and 95% confidence intervals for the odds ratios. Overall tests for multi-category variables (e.g., age group, sex, race, education) are shown before their individual contrasts. Statistical significance at p < .05 is indicated by p-values less than .05; p-values less than .001 are indicated as <.001.
